# Functional Properties and Sensory Quality of Kombucha Analogs Based on Herbal Infusions

**DOI:** 10.3390/antiox13101191

**Published:** 2024-09-30

**Authors:** Marta Czarnowska-Kujawska, Joanna Klepacka, Małgorzata Starowicz, Patrycja Lesińska

**Affiliations:** 1 Department of Commodity and Food Analysis, The Faculty of Food Sciences, University of Warmia and Mazury in Olsztyn, 10-726 Olsztyn, Poland; klepak@uwm.edu.pl (J.K.); patrycja.lesinska@student.uwm.edu.pl (P.L.); 2 Department of Chemistry and Biodynamics of Food, Institute of Animal Reproduction and Food Research of Polish Academy of Sciences, 10-748 Olsztyn, Poland; m.starowicz@pan.olsztyn.pl

**Keywords:** SCOBY, mint, nettle, blackcurrant leaves, organic acids, phenolic compounds, antioxidant activity, minerals

## Abstract

Traditionally, kombucha is produced by the fermentation of black or green tea infusions with the use of SCOBY (Symbiotic Culture of Bacteria and Yeasts). However, SCOBY exhibits the ability to ferment other substrates as well, which can be used to create novel products with new sensory and health-promoting properties. This paper investigates the antioxidant activity, chemical composition, and sensory properties of mint, nettle, and blackcurrant leaf-based kombucha analogs. It has been demonstrated that the fermentation process with SCOBY significantly influenced (*p* ≤ 0.05) sugar, organic acids, and mineral contents, with the increase in iron, magnesium, and calcium amounts in all tested herbal kombucha. The study shows that the type of herb infusion has a significant influence on the parameters associated with antioxidant potential. The fermentation with SCOBY resulted in an increase in antioxidant activity as measured by the superoxide anion radical (O_2_^•−^) inhibition of all three tested herbal infusions, with the greatest changes observed in nettle kombucha. Herbal kombucha was characterized by significantly increased total phenolic content as determined by Folin’s reagent and a changed phenolic compound profile by LC-MS/MS (liquid chromatography with tandem mass spectrometry) in comparison to nonfermented infusions. Very high sensory scores were achieved for fermented mint and blackcurrant-based kombucha.

## 1. Introduction

With changing dietary trends and growing consumer awareness, the food market has developed dynamically. Satisfying basic physiological needs is no longer the most important criterion when choosing food products. Consumers are searching for food stuffs, including fermented products, that are new and innovative, with increased health-promoting ingredients and sensory appeal [1,2]. The fermentation process itself has been used in food production for centuries. The process of fermentation and the changes in the food composition it causes make it possible to extend shelf life, increase safety, and, most importantly, allow the development of products with unique properties, which are not only sensory but also beneficial for health [1,3].

Kombucha is a beverage made from black or green sweetened tea infusions fermented with so-called “tea fungus”, which is a *Symbiotic Culture of Bacteria and Yeasts* (SCOBY). The choice of tea as the raw material is significant for the final properties of the obtained fermented beverage due to its antioxidant activity, mainly relating to the presence of polyphenols and also the content of certain minerals, especially manganese [4]. Many authors [2,5,6,7] indicated green tea, which is minimally processed, as the best source of polyphenols because the enzyme that causes their oxidation, polyphenol oxidase, is inactivated in leaves as soon as they are harvested. Black tea is fully oxidized, and the oxidation process leads to a change in the form of the phenolic compounds and also to a decrease in their content. The antioxidant activity of tea infusions also depends on their preparation, i.e., the temperature of the water used and the number and time of extraction [4,8,9]. Apart from substrate nutrition value, the bioactive and chemical composition of kombucha also depends on the fermentation process parameters and microbes applied [2].

Increasingly, traditionally used raw materials in kombucha production, such as tea, are being replaced by other alternative substrates, e.g., fruits, vegetables, milk, or agro-waste. The use of unconventional raw materials makes it possible to obtain kombucha analogs with altered sensory and physicochemical properties [2,10,11,12]. Recent studies have proven that the fermentation process carried out with the use of SCOBY increased the nutritional value of beverages, as it led, among other things, to an increase in the level of phenolic compounds and, thus, to an increase in antioxidant activity that results from the presence of these compounds [2,12,13,14]. Although most often described in the literature, the changes occurring within the polyphenols in kombucha-type beverages are not the only ones. Bacteria and yeasts involved in the fermentation process generate many more beneficial compounds such as organic acids, vitamins, minerals, and hydrolytic enzymes [1,11,14,15]. By fermenting with SCOBY non-typical substrates, which contain a wide range of various bioactive compounds, the antioxidant, antimicrobial, anti-inflammatory, and anti-hypertension properties of kombucha can be improved [1,2,12]. However, the chemical composition and resulting health-promoting properties of the new kombucha analogs need to be known and monitored as they are novel products.

Herbs are widely used as food, spices, and flavoring agents. Some of them, like mint (*Mentha piperita*), show high biological activity and beneficial medicinal effects thanks to their high content of bioactive compounds, among which polyphenols play an important role [16,17]. The main constituents of infusions prepared from mint leaves as well as the alcoholic extracts used, e.g., for medicinal purposes, are non-volatile, polar phenylpropanoids, including mainly phenolic acids (e.g., rosmarinic acid and chlorogenic acid) and also flavonoids such as apigenin and luteolin [16]. The recent study of Staszowska-Karkut et al. [18] confirmed that blackcurrant leaves (*Ribes nigrum folium*) are a rich source of phenolics with high antioxidant activity and anticancer properties. Among phenolic compounds, quercetin and its derivatives, as well as myricetin, dominate [18]. Apart from polyphenols, among which rutin and sinapic acid are predominant, nettle leaves (*Urticae folium*) are rich in terpenoids, carotenoids, fatty acids, essential amino acids, chlorophylls, vitamins, carbohydrates, sterols, polysaccharides, isolectins, and minerals [19,20,21]. Another advantage of herbs is their widespread availability; they can be purchased in herbal stores, supermarkets, and through Internet sales, which means that the consumer can easily prepare herbal-based kombucha at home.

In the present study, three types of kombucha were prepared using different herbs, mint, nettle, and blackcurrant leaves, all obtained on the market for retail sale. The choice of herbs was dictated by their health-promoting properties and the sensory qualities of the infusions and the kombucha prepared from them, as well as their easy accessibility for consumers. The aim was to perform a comparative study on the antioxidant, physicochemical, and sensorial properties of the obtained herbal kombucha analogs. In addition to the results of antioxidant activity and the results of the phenolic compounds, the content of sugar, organic acids, and selected micro and macro elements are presented in this work.

## 2. Materials and Methods

### 2.1. Materials

SCOBY, with a diameter of 9 cm with the addition of a starter (100 mL), was purchased from an online store (Łódź, Olsztyn, Poland) specializing in the sale of various types of microbiological starters. Leafy green tea and sucrose were obtained from a local market in Olsztyn, Poland. Dried leaves of mint (*Mentha piperita folium*), nettle (*Urticae folium*), and blackcurrant (*Ribes nigrum folium*) were purchased from a herbal shop in the area of Olsztyn, Poland.

### 2.2. Chemicals and Reagents

Water was purified in the Mili-Q system (Millipore; Vienna, Austria), trifluoroacetic acid (TFA), acetonitrile, methanol (MeOH), water, and formic acid (FA) of MS-grade purity was purchased from Merck (Darmstad, Germany). _D_-glucuronic acid was bought from Merck (Darmstad, Germany), while acetic acid, citric acid, and succinic acid from Supelco (Bellefonte, PA, USA). Kits for water-soluble (ACW) and lipid-soluble antioxidants (ACL) for the photochemiluminesce (PCL) method were purchased from Analytik Jena (Jena, Germany), whereas 2,2′-diphenyl-1-picrylhydrazyl (DPPH) and gallic acid were obtained from Sigma Aldrich Chemical Co. (St. Louis, MO, USA). Phenolic acids (caffeic, chlorogenic, *p*-coumaric, ferulic, gentisic, hippuric, *p*-hydroxybenzoic, *m*-hydroxyphenylacetic, protocatechuic, salicylic, sinapic, syringic, and vanillic acids) and flavonoids (epicatechin, kaempferol, myricetin, and rutin) standard compounds were obtained from Extrasynthese (Genay, Rodan, France) and Merck (Darmstad, Germany). Hydrated lanthanum chloride (Cl_3_La • 7H_2_O), used in mineral content determination, was purchased from Merck (Darmstadt, Germany), while ammonium molybdate VI, sodium sulfate IV, and hydroquinone were purchased from “POCH” S. A. (Gliwice, Poland).

Other chemicals used in the experiments were at least of analytical grade and were purchased from Merck (Darmstadt, Germany) and “POCH” S. A. (Gliwice, Poland).

### 2.3. Preparation of Fermented Kombucha Analogs

At first, the green tea-based starter was prepared, which was used for further herbal-based kombucha production. 8 g of green tea leaves were placed in a sterilized glass jar, poured with 1 L of hot water (90 °C), and infused for 6 min. Tea leaves were then strained, 100 g sugar was added, and the whole was mixed. After cooling the drinks to room temperature (21 °C), 100 mL of the previously purchased starter and 30 g of SCOBY were added, and the jar was secured with a cotton cloth to protect the liquid from contamination and left to ferment for seven days.

When the green tea kombucha was ready, the preparation of fermented SCOBY herbal infusions started. Moreover, 8 g of mint, nettle, and blackcurrant leaves were weighed out and placed in separate sterilized glass jars, and each was poured with 500 mL of hot water (90 °C). The whole mixture was infused for 6 min. The obtained infusions were strained through a sieve and then transferred to a large, sterilized glass jar. Another 500 mL of boiled water, together with 100 g of sugar, were added to the still-warm solutions and mixed. 100 mL of the previously prepared green tea-based kombucha and SCOBY (30 g) were added to cooled infusions (room temperature). Jars were covered with a sterile cotton cloth, and the liquids were left to ferment for seven days. Then, the fermented infusions were subjected to sensory evaluation, and samples were collected for further chemical analyses.

### 2.4. pH Determination

The HI98100 pH meter (Hanna Instruments; Woonsocket, RI, USA) was used for the pH measurement of tested beverages during fermentation.

### 2.5. Organic Acid Analysis

10 mL of the infusion was transferred to a 50 mL volumetric flask and made up to the mark with 20 mM KH_2_PO_4_ (pH 2.4) and shaken for 1 h. Then, the samples were filtered (0.22 µM) directly into HPLC vials. Organic acids (acetic acid, citric acid, succinic acid and _D_-glucuronic acid) were analyzed using HPLC equipped with UV detector (Shimadzu Nexera-i LC-2040 C plus; Shimadzu Co.; Kyoto, Japan) set at 214 nm and Phenomenex C18 column (4.6 × 250 mm, 5µM; Phenomenex; Torrance, CA, USA) [15]. The mobile phase was a mixture of 20 mM KH_2_PO_4_ (pH 2.4) and methanol (97:3). The flow rate and column temperature were maintained at 0.7 mL/min and 25 °C, respectively.

### 2.6. Sugar Content Analysis

The total sugar content of the analyzed beverages was measured using a digital refractometer (HI96801, Hanna Instruments, Woonsocket, RI, USA). A few drops of the infusions were applied directly to the built-in prism of the refractometer. The result was obtained after 2 s with a measurement accuracy of ±0.2%.

### 2.7. Analysis of Antioxidant Activity

The photochemiluminesce (PCL) method described by Zieliński et al. [22] was used to determine the ability of kombucha drinks to scavenge the superoxide anion radical (O_2_^•−^). Samples were diluted either with methanol in ACL or buffer in case of ACW. Analysis was carried out with the use of Photochem^®^ apparatus (Analytik Jena, Jena, Germany). The results were calculated on the basis of the Trolox standard curve (R^2^ = 0.9997 in ACW; R^2^ = 0.9999 in ACL) and presented as µmol Trolox eq./g of sample.

The ability of tested beverages to neutralize the DPPH (2.2-diphenyl-1-picrylhydrazyl) radical was checked with the method previously described by Klepacka et al. [4]. The determination was performed on the basis of colorimetric changes in the concentration of the stable DPPH radical in relation to the control sample. Moreover, 1 mL of methanolic tested beverage solution was introduced to 4 mL of 0.1405 mM methanolic DPPH solution. Absorbance was measured at a wavelength of λ = 517 nm (Thermo Scientific spectrophotometer Helios Zeta UV-VIS, Madison, WI, USA) at the beginning and after 20 min of the reaction against a blank sample (4 mL of DPPH solution + 1 mL of methanol) at room temperature without light. The ability of the tested extracts to counteract the oxidation reaction was calculated from the formula:

% inhibition = 100 − {[(Aw − A0) × 100]/Ak}

where Aw—absorbance of the tested extract; A0—absorbance of the zero sample; Ak—absorbance of the control sample (with a synthetic DPPH radical).

### 2.8. Determination of Total Content of Phenolic Compounds

The total phenolic compound content (TPC) in the analyzed beverages was determined by a spectrophotometric method using the Folin–Ciocalteu reagent with gallic acid as a standard, according to Klepacka et al. [4]. Moreover, 0.04 mL of tested beverage and 6 mL of redistilled water were briefly added to 0.5 mL of Folin–Ciocalteu reagent. The whole was mixed, and after 3 min, 1.5 mL of 20% (*w*/*w*) aqueous sodium carbonate solution was added and made up to the mark with redistilled water. The samples were left in the dark for 1 h, and then the absorbance was measured at 765 nm against the blank (Thermo Scientific Helios Zeta UV-VIS, Madison, WI, USA). The results were expressed as gallic acid equivalent with a reference curve plotted for this acid:

𝑦 = 0.0223𝑥 − 0.1916, R^2^ = 0.990

### 2.9. Determination of Phenolic Compounds by HPLC

The profile and content of phenolic acids and flavonoids were determined following the method established previously by Płatosz et al. [23]. The kombucha drinks were vortexed for 1 min and sonicated for another 1 min (VC 750, Sonics & Materials Inc., Newtown, CT, USA), and finally centrifuged (20 min, 13,200× *g*, 4 °C; Centrifuge 5415 R, Eppendorf, Hamburg, Germany).

In the last step, samples were analyzed using an LC-MS system equipped with micro-HPLC (LC-200, Eksigent) and a degasser, two binary pumps, and an autosampler. The LC-MS system was coupled with a QTRAP 5500 detector (AB Sciex, Vaughan, ON, Canada) consisting of a triple quadrupole, ion trap, and electrospray ionization (ESI) ion source. 5 µL of pre-treated samples was injected into a HALO C18 column (0.5 mm × 100 mm × 2.7 μm, Eksigent, Vaughan, ON, Canada) kept at 45 °C. The flow rate was 15 μL/min with solvent A—water with 0.1% FA and solvent B—0.9% FA in a solution of acetonitrile with 9.1% methanol. The gradient started from 1% B for the first min. Next, the mobile phase composition was changed by increasing B to 90% (1 to 4 min) and returned to starting conditions in 0.5 min, keeping the re-equilibration at 1% B for 1 min. Data were collected in negative ion mode. The curtain gas was set to 20 L/min, and the following settings were used: collision gas: ion spray voltage: 5300 V; temperature: 350 °C; 1 ion source gas: 35 L/min; 2 ion source gas: 30 L/min; declastering potential: 100 V; entrance potential: 10 V; collision energy: 40 eV; and collision cell exit potential: 20 V. The identity and quantity of phenolic acids and flavonoids were confirmed by matching the experimental MS/MS spectra to MS/MS spectra from databases and fragmentation spectra and retention times (Multiple Reaction Monitoring, MRM) obtained for the standards (Table S1 in Supplementary Materials). The linear calibration curves of external standards had correlation coefficients of 0.979−1.000 (Table S2 in Supplementary Materials). The results were expressed in µg/mL.

### 2.10. Mineral Content Analysis

The method for selected mineral content determination was directly adopted from Klepacka et al. [4] and Czarnowska-Kujawska et al. [24]. The analysis of copper (Cu), manganese (Mn), iron (Fe), zinc (Zn), magnesium (Mg), and calcium (Ca) was carried with the use of flame atomic absorption spectrometry (acetylene—air flame) equipped with a Thermo iCE 3000 Series atomic absorption spectrometer (Madison, WI, USA) with a Glite data station, deuterium lamp as a background correction, and appropriate cathode lamps. Elements were determined at the following wavelengths: Cu—324.8 nm, Mn—279.5 nm, Fe—248.3 nm, Zn—213.9 nm, Mg—285.2 nm, and Ca—422.7 nm. Determination of sodium (Na) and potassium (K) was carried with the emission technique (acetylene-air flame) using an atomic absorption spectrometer Thermo iCE 3000 Series (Waltham, MA, USA), equipped with a Glite data station, operating in an emission system. The wavelengths were set at 589.0 nm for Na and at 766.5 nm for K determination.

The determination of phosphorus (P) content was carried out by the colorimetric method with ammonium molybdate, sodium sulphate (IV), and hydroquinone. Ammonium molybdate was transformed into phosphomolybdates, which were then reduced to phosphomolybdenum blue using sodium sulfate and hydroquinone. Analysis was performed using a VIS 6000 spectrophotometer (KRÜSS-OPTRONIC, Hamburg, Germany) set at 610 nm. The content of all minerals was calculated using standard curves prepared individually for each of them.

The concentrations of the standard solutions of the individual micro- and macroelements used formed the determination range of the analytical method used in the experiment, which was characterized by the linearity of the calibration curves. The limits of the measurement range adopted for the individual elements determined the equation and the coefficient of the calibration curve, which are shown in Supplementary Materials (Table S3). In accordance with the recommendations of the validation procedures, the limits of quantification of the selected elements were determined on the basis of the analyses performed: Cu (0.05 mg/kg), Mn (0.05 mg/kg), Fe (0.2 mg/kg), Zn (0.05 mg/kg), mg (0.05 mg/kg), Ca (0.5 mg/kg), Na (0.5 mg/kg), K (2.0 mg/kg), and P (0.4 mg/kg).

### 2.11. Sensory Analysis

The tested kombucha beverages derived from herbal infusions were sensory evaluated using the differential profiling method by a trained team of ten panelists from the Faculty of Food Sciences of the University of Warmia and Mazury in Olsztyn. Sensory analysis was carried out under controlled conditions in a sensory analysis laboratory. The panelists’ task was to mark on a bipolar scale how much a specific feature of the tested sample differs from the standards, which were unfermented test materials. A deviation scales from −3 (the lowest intensity of the tested sensory characteristic) to +3 (the highest intensity of the tested sensory characteristic), where 0 corresponded to the quality of standard sample, were used. The scores for color, clarity, taste, odor, and overall acceptability were given by each member of the team. All individual participants took part in the study voluntarily, knowing its purpose and scope. Participants gave informed consent that they were aware their responses were confidential. They were able to withdraw from the survey at any time without giving a reason. Prior to analysis, panelists completed a written consent form for future publication of the obtained sensory evaluation results. A template of this consent is attached in Supplementary Materials.

### 2.12. Statistical Analysis

All results were presented as mean ± standard deviation of three replicates. The level of statistical significance among the means was analyzed by one-way ANOVA using Statistica software version 13.2 (StatSoft; Cracow, Poland). The significance of differences was analyzed at a significance level of *p* ≤ 0.05.

## 3. Results and Discussion

### 3.1. The Changes in pH, Organic Acids, and Sugar Contents during Fermentation

**Figure 1:**
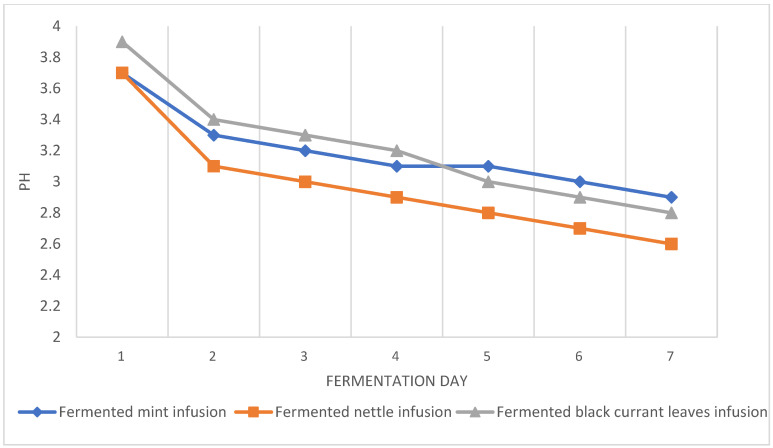
pH changes of tested beverages.

With maintaining the same parameters of brewing herbal leaves, the active acidity (pH value) of individual infusions was similar and ranged from 3.7 for mint and nettle infusions to 3.9 for blackcurrant leaf infusion (Figure 1). The pH of all tested herbal kombucha analogs decreased with fermentation time. At the end of fermentation, the pH of fermented mint, nettle, and blackcurrant leaves beverages were 2.9, 2.6, and 2.8, respectively. The greatest decrease in pH was observed during the first days of the fermentation process, which is in agreement with other authors’ studies [13,14,15].

The observed decrease in pH value results from the action of bacteria and yeast, which metabolize sucrose to numerous organic acids [13,14,15]. With increased concentration of organic acids, the pH of kombucha beverages decreases within fermentation time. However, it should be noted that based on an FDA (Food and Drug Administration) recommendation, the lowest acceptable pH of consumed drinks should not be lower than 3.0 as the increased consumption of drinks with very low pH can cause negative effects on the digestive system [11,13,25]. This indicates the need to continuously monitor the fermentation of kombucha beverages by systematically determining their acidity and stopping the process when their pH is close to 3.

The amounts of produced organic acids in fermented herbal kombucha analogs are presented in Table 1. Acetic acid was the major organic acid found in beverages after fermentation, with the concentration varying from 3.43 g/L in fermented blackcurrant leaves infusion to 6.47 g/L in fermented nettle infusion. During the fermentation process, sucrose is hydrolyzed to glucose and fructose by yeast invertase, and ethanol is produced. In the next stage, ethanol is oxidized to acetic acid by acetic acid bacteria [15,26]. In the case of citric acid, the significant (*p* ≤ 0.05) increase in its content was only shown for fermented nettle infusion. However, its concentration in all tested kombucha was very low. All types of tested herbal infusions were very good substrates for succinic acid production, with the highest, almost fivefold, increase in fermented mint infusion (0.19 g/L). Although Jayabalan et al. [15], in the study on green and black tea kombucha fermentation, reported glucuronic acid as the next major organic acid after acetic acid, our research did not confirm this. This proves the essential effect of used substrate for kombucha production on the chemical composition of fermented beverages.

Apart from the changes in pH and organic acid concentration, a significant (*p* ≤ 0.05) decrease in sucrose was also observed in tested fermented herbal beverages (Table 1). Sucrose is considered the most commonly used carbon source in kombucha production [27]. As the fermentation process proceeds, the sugar content decreases [13,14]. However, according to Malbaša et al. [28], a significant amount of sucrose is not fermented. The lowest sugar consumption of 7%, in comparison to the nonfermented sweetened infusions, was observed in kombucha derived from blackcurrant leaves. This beverage was also characterized by the lowest acetic acid content of 3.43 g/L from all tested herbal kombucha.

### 3.2. Antioxidant Activities

The antioxidant activity of herbal kombucha analogs was measured by determining the ability of the antioxidants to quench two types of radicals, superoxide anion radicals (O_2_^•−^) determined with the PCL method and DPPH radicals determined spectrophotometrically. Regardless of the method used, mint infusions were characterized by the highest antioxidant activity among nonfermented beverages (Table 2). Their total antioxidant activity measured by the PCL method was 9.14 µmol Trolox/mL, and determined by the degree of quenching of the DPPH radical was 80.2%. Nettle nonfermented infusion was characterized by the lowest antioxidant properties with several times lower values for PCL and DPPH, 0.79 µmol of Trolox/mL and 15.2%, respectively. Similar differences in the antioxidant activity of mint and nettle extracts were also reported by Masłowski et al. [29]. Differences in the level of antioxidant activity of various herbs were confirmed by many authors, indicating that not only species and variety factors are important but also the harvesting conditions, processing method, and parameters, as well as storage [30,31,32,33,34,35].

For both tested unfermented and fermented with SCOBY beverages, a higher level of lipid-soluble antioxidants (ACL) was observed than water-soluble compounds (ACW) (Table 2). This observation was confirmed by the research of Masłowski et al. [29], who reported that the main antioxidants of herbs are phenolic compounds, which dissolve much better in organic solvents (ACL fraction) than in water (ACW fraction). Thus, to extract these components from herbs, the authors recommended using mixtures of organic and inorganic solvents (e.g., water–methanol or water–ethanol), which resulted in an 11-fold better extraction of phenols from mint and more than 40-fold higher extraction from nettle, compared to using water alone. Importantly, the antioxidant activity of herbs results also from the presence of vitamin C or carotenoids, as well as many other components contained in essential oils [36]. Therefore, the total antioxidant activity of various herbal raw materials will depend on the proportion of these components and their solubility in the reagents used for extraction [29,32,37,38].

The fermentation with SCOBY resulted in an antioxidant activity increase in all three tested herbal infusions, which was determined by comparing unfermented and fermented drinks. The greatest changes were observed in nettle kombucha, in which antioxidant activity measured by the PCL method for ACW increased almost tenfold (from 0.16 to 1.08 µmol Trolox/mL), and for ACL, it increased about fourfold (from 0.62 to 2.31 µmol Trolox/mL) in comparison to the nettle infusion before fermentation (Table 2). A greater increase in antioxidant activity associated with the presence of water-soluble components was also observed in mint-based kombucha. The content of ACW under fermentation doubled from 2.45 to 5.17 µmol/mL, while the level of ACL increased to a lesser, but still statistically significant (*p* ≤ 0.05) degree from 6.70 to 8.13 µmol/mL. The smallest changes for ACW and ACL under fermentation were observed for kombucha derived from black currant leaves. A similar rate of increase in antioxidant activity of herbal infusions treated with SCOOBY was indicated by other authors [39,40], while Kilic and Sengun [41] reported that depending on the method of infusions’ preparation, and especially the type and amount of sweeteners added, the antioxidant activity during kombucha fermentation may also decrease. Saritas et al. [42] confirmed that the rate and degree of changes in antioxidant activity of fermented food depends on many factors, among which the most important are the chemical composition and properties of the fermented products as well as the type and conditions of fermentation. There are many literature sources related to the properties of kombucha fermented tea, e.g., Wang et al. [43] and Zhou et al. [14] showed that using tea fungus had a much greater impact on the increase in antioxidant activity of green tea infusions compared to black tea which might result from the different methods of leaves production. Emiljanowicz and Malinowska-Pańczyk [32] pointed out the possibility of obtaining kombucha-type drinks with various health-promoting properties from many alternative raw materials, among which, in addition to herbs, they indicated vegetable pulp, fruit juices, soybean whey, banana peel or milk. In our previous study on kombucha analogs based on cow’s milk and selected plant-based drinks [44], we showed that under fermentation with SCOBY, antioxidant activity can increase on average 3–4 times and, in some cases, even 28 times. Many authors link the positive effect of kombucha fermentation on antioxidant activity with the duration of this process [30,36,39,45]. Ahmed [30] and Wang et al. [43] associated these changes with chemical transformations of phenolic compounds, which under the influence of enzymes produced during fermentation, can be hydrolyzed to more easily detectable low-molecular-weight compounds and can be further released from insoluble combinations in which they occurred with other components. According to Essawet et al. [38], higher antioxidant activity in kombucha-type tea beverages resulted from an increase in polyphenols but also tea fungus metabolites, such as vitamins and organic acids. Furthermore, Zhou et al. [14] suggested that fermentation conditions, including pH and microbial activity, caused destruction of the cellular structure of the used raw materials, which promoted additional transfer of water-soluble antioxidant compounds into the fermented beverages. This observation may also explain the mentioned greater increase in water-soluble antioxidant compounds than in lipid-soluble compounds (Table 2). Additionally, Jakubczyk et al. [13] reported the unique influence on the observed changes in the reduction of the dominant role of yeast and the intensive development of lactic acid bacteria on day seven of tea kombucha fermentation.

In our study, the degree of change in antioxidant activity under the influence of the fermentation process measured by DPPH radical scavenging was much lower than that of the superoxide anion radical (O_2_^•−^) inhibition measured by the PCL method. For example, in fermented nettle infusions, PCL values increased fourfold (from 0.79 to 3.39 µmol/mL), while the degree of DPPH radical scavenging increased just twofold (from 15.2 to 33.6%). Interestingly, in mint infusions, due to the fermentation process, antioxidant activity measured by both ACW and ACL increased, while the level of DPPH radical scavenging decreased from 80.2 to 74.1%. The observed correlations are probably due to the different mechanisms of antioxidant activity of the components present in the tested beverages and their different activities in inhibiting O_2_^•−^ and DPPH radicals, as confirmed by other authors [12,13,14,34,39,43].

### 3.3. TPC Values and HPLC Profile of Phenolic Compounds

In the tested beverages, total phenolic content (TPC) by Folin’s reagent and phenolic compounds profile by LC-MS/MS has been determined. Generally, it can be summarized that SCOBY fermentation of tested herbal beverages increased significantly (*p* ≤ 0.05) TPC values (Table 3). The increasing trend of TPC can be explained as a formation of new metabolites and also as the consequence of reveling compounds from plant matrix. That might compensate for the degradation of some phenolic compounds during fermentation [46]. This is in line with Leonard et al. [46], who gathered several studies that proved higher bioaccessibility of phenolic compounds from plants after fermentation with different microorganisms. The highest TPC has been obtained for SCOBY fermented mint infusion. However, the highest increase (~85%) of TPC value after fermentation has been observed for nettle infusion, then for mint and blackcurrant, ~59 and 19%, respectively. The demonstrated trends are in accordance with the relationships discussed in Chapter 3.2, which confirms that the main antioxidants of the analyzed beverages are phenolic compounds. The TPC for fermented products can be ordered as follows: mint (102.2 ± 0.75 mg/100 mL) > blackcurrant (82.0 ± 2.18 mg/100 mL) > nettle infusions (45.4 ± 0.97 mg/100 mL). The TPC value of fermented mint correlates with the highest antioxidant activity measured with the ACL. Gan et al. [47] also reported increased TPC value in plant-based milks inoculated with *Lactobacillus plantarum* WCFS1 and its correlation with the antioxidant activity of lipophilic fractions.

Thirteen phenolic acids have been determined in all types of SCOBY fermented and nonfermented beverages (Figure S1 in Supplementary Materials). The content of phenolic acids significantly (*p* ≤ 0.05) increased in fermented mint and nettle infusions. Furthermore, syringic acid has been identified as a major phenolic acid in fermented infusion, and its content significantly (*p* ≤ 0.05) increased in all infusions after fermentation with a SCOBY culture. Syringic acid highly contributed to the sum of phenolic acids of SCOBY fermented beverages (approx. 93% in mint, 94% in nettle, and 85% in blackcurrant infusions). The highest content of syringic acid has been found in fermented mint and nettle infusions (48.61 ± 0.59 µg/mL and 25.19 ± 1.15 µg/mL, respectively). Therefore, it was found that the fermentation positively affects the formation of syringic acid. As blackcurrants are a rich source of anthocyanins, a higher content of syringic acid could be related to the transformation of specific anthocyanins (e.g., malvidin 3-glucoside) as the effect of the fermentation process [46]. The opposite effect has been observed in the case of *p*-coumaric acid; its content decreased in all samples, the most significantly in blackcurrant beverages after fermentation. Tsakni et al. [48] also identified some of the same bioactive compounds in Greek mint, e.g., caffeic, ferulic, *p*-coumaric, and hydroxybenzoic acids. However, their amount differentiated from our results. Such differences could be related to the various geographical and botanical origins of herbs. Elez Garofulić et al. [19] also found that the temperature of the extraction method could influence the content and profile of phenolic compounds of nettle.

Kaempferol, epicatechin, myricetin, and rutin, from the flavonoid group of compounds, have been detected in tested infusions (Table 3, Figure S1 in Supplementary Materials). Mostly, epicatechin has been detected in nonfermented infusions. The significant (*p* ≤ 0.05), at least two-fold, degradation of epicatechin was observed after SCOBY fermentation in each fermented product. Apart from epicatechin, infusions of nettle and blackcurrant rutin have also been identified. In some other studies, rutin was noted as a main flavonoid in nettle [19,49]. We observed that fermentation caused the degradation of rutin, possibly due to quercetin whose amount was too low to quantify. Such effects of phenolic compound transformations have been discussed in detail by Leonard et al. [46]. Based on the obtained results for tested herbal kombucha analogs, it can be concluded that phenolic acids, rather than flavonoids, are the main contributors to the overall bioactivity of the beverages tested.

### 3.4. Contents of Micro and Macroelements

Contrary to numerous scientific reports on the antioxidant potential of various kombucha beverages, there is little data on the mineral content of these drinks. The data need to be supplemented, given the important role played by both macronutrients, which occur in larger quantities in the body, and microelements, present in trace amounts, for proper functioning of the human body [50,51]. Therefore, it was particularly important in our work to indicate the direction of changes in individual element contents under fermentation with SCOBY.

Nonfermented infusions were characterized by different microelement contents (Table 4) depending on the used herb. High variations between herbs infusions were observed in manganese (from 5.23 µg/100 mL in nettle infusion to 12.66 µg/100 mL in blackcurrant leaves infusion) and zinc (12.78 µg/100 mL in blackcurrant leaves infusion to 22.60 µg/100 mL in nettle infusion) contents. The nonfermented mint infusion contained nearly four times as much iron (6.10 µg/100 mL) as the other two infusions (1.38 µg/100 mL and 1.67 µg/100 mL in nettle and blackcurrant leaves infusions, respectively). The fermentation with SCOBY caused significant (*p* ≤ 0.05) changes in all analyzed microelement contents. Herbal kombucha was characterized by the highest contents of zinc and manganese, which is in agreement with data reported by Jakubczyk et al. [50] for kombucha prepared with black, red, green, and white tea and by Ivanišová et al. [33] for black tea-based kombucha. In all tested beverages, a significant decrease, even two times in nettle-based kombucha, in copper content was noticed after fermentation. Conversely, for iron, a significant increase—even twofold—in fermented nettle beverages was observed in all analyzed samples after fermentation. In the case of manganese content, the only increase, more than 50%, was noticed in fermented nettle infusion, while for zinc content, more than a 25% increase was observed in blackcurrant leaf-based kombucha. In contrast, for kombucha derived from black tea, Ivanišová et al. [33] observed the increase during fermentation of all—iron, manganese, zinc, and nickel, the last of which was not analyzed in our study.

Among macroelements, the lowest variations between different herbal infusions used for kombucha production were reported for magnesium (2.40 mg/100 mL–3.19 mg/100 mL), sodium (2.19 mg/100 mL–2.48 mg/100 mL), and phosphorus (3.85 mg/100 mL–5.85 mg/100 mL) contents (Table 5). Much higher calcium content, compared to two other tested infusions, was found in nettle infusion (13.20 mg/100 mL), and potassium was found in mint infusion (22.16 mg/100 mL). As with microelements, fermentation of herbal infusions with SCOBY resulted in significant (*p* ≤ 0.05) changes in the content of all analyzed macroelements. Fermentation with SCOBY caused a significant decrease in phosphorus content in all tested infusions, with the highest 19% loss in mint-based kombucha. All fermented beverages were characterized by increased magnesium (more than 55% increase in nettle and blackcurrant leaf-based kombucha) and calcium (approx. 10% increase) contents. A significant 11% sodium increase was observed in fermented mint and blackcurrant leaf infusions in comparison to samples before fermentation. In the case of potassium, an increase in content was noticed only in nettle kombucha, approx. 10%.

In our study, we showed that mineral contents in tested kombucha analogs were influenced by the raw material (mint, nettle, and blackcurrant leaves) used for their production. The same was observed by Jakubczyk et al. [50], who additionally pointed to the day of fermentation as a determinant of changes in mineral content. Certainly, the final content of minerals in SCOBY fermented beverages is influenced by these nutrients’ original content in the infusions, which is the substrate for kombucha production. In the case of tea infusions, Klepacka et al. [4] indicated numerous factors such as tea variety, location, agriculture method, and practices, including method of leaf cultivation, harvesting, storage, and transport, that can affect micro and macroelement contents. In our experiment, fermentation with SCOBY significantly (*p* ≤ 0.05) increased iron, magnesium, and calcium contents in all tested herbal-based kombucha, which, considering the important role of these elements in the human organism [51], is important and beneficial for a nutritional point of view. The reported increase in individual elements in tested herbal kombucha is most likely the effect of collaboration between yeasts and bacteria during fermentation. The symbiotic culture of bacteria and yeast utilizes substrates in different metabolic pathways to generate several efficient metabolites, which, apart from polyphenols, organic acids, ethanol, vitamins, and enzymes, includes also minerals [11,36,50,52]. As for the observed decrease in the content of certain minerals in analyzed herbal kombucha analogs, these elements could be used for the microorganisms’ own metabolism as a substrate to start the fermentation process or for secondary metabolites, such as vitamins and polyols, synthesis [50,53]. The observed copper decrease in all tested herbal kombucha could be explained with detoxifying SCOBY properties, thanks to the ability to accumulate and bind heavy metals on their cellular structure [33]. Acting as biosorbents, kombucha microorganisms were previously reported to be very effective in removing heavy metals (arsenic, chromium, and copper) in the beer brewing process [54].

### 3.5. Evaluation of Sensory Properties

Literature data report that kombucha is generally fermented from ten to even fourteen days [11]. In our study, around ten days of fermentation, the taste of herbal kombucha was too intense, sour, and bitter to be accepted. Sourness results from the presence of acetic acid in kombucha [11], while bitterness results from the presence of polyphenols and is often masked by increasing the perceived sweetness level [1,15]. However, during the first days of fermentation, other authors [15] have observed that a decrease in the content of polyphenols, such as epicatechin, epigallocatechin, and gallate derivatives, contributed to the bitterness in kombucha because, as the sugar content decreased during fermentation, the sweetness decreased, and therefore the sensation of bitterness might be too intense. For this reason, in our study, fermentation process was carried out for no more than seven days.

**Figure 2:**
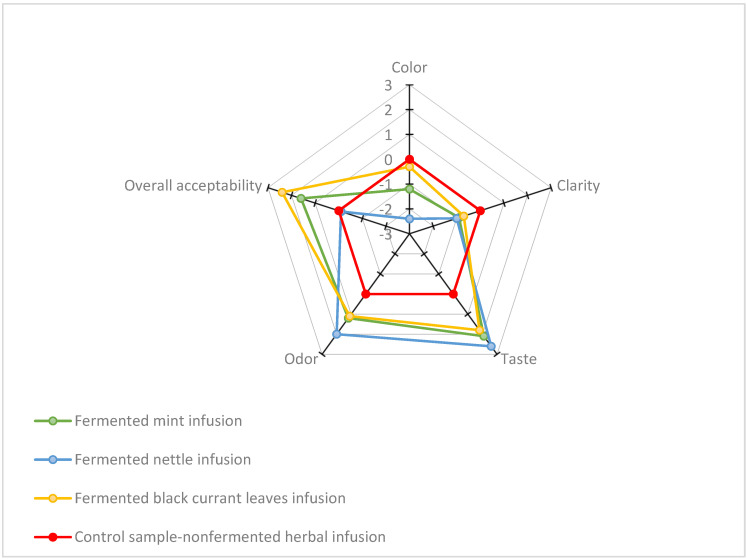
Sensory evaluation of tested herbal kombucha analogs.

The sensory attributes of color, clarity, taste, odor, and overall acceptability for three types of herbal kombucha in comparison to the control sample—nonfermented herbal infusions were evaluated. The results are presented in Figure 2. During the fermentation process, the kombucha color changed depending on the substrate used for its production. For instance, green tea-based kombucha exhibits a light-brown color, while kombucha produced from black tea is dark-brown [11,55]. In our study, seven-day fermentation caused color changes in all tested herbal beverages. Their color has become visibly lighter. The brightest straw color (−2.4) showed the nettle-based kombucha in comparison to the intense, dark color of the infusion before fermentation. The color changes in beverages after SCOBY fermentation may result from breaking down catechins, theaflavins, and thearubigin to simpler forms under the influence of enzymes produced by the acetic acid bacteria and yeast involved in the fermentation process [15]. All fermented beverages were less clear compared to unfermented herbal infusions. Both the taste and aroma of herbal infusions became more intense after SCOBY fermentation, with the highest scores for the nettle leaf-based kombucha, 2.6 for taste and 2.0 for odor. The increase in flavor intensity of herbal infusions after SCOBY fermentation is most likely due to the ethanol and acetic acid produced during the process [56]. In terms of overall acceptability, the highest ratings were given to black currant (2.4) and mint leaf (1.6)-based kombucha, most likely due to their delightful, refreshing taste and pleasant color.

## 4. Conclusions

Fermentation with SCOBY alternative substrates like herbal infusions has shown results that make them interesting candidates for the domestic preparation of kombucha-type drinks due to their composition and functionality. Fermentation with SCOBY influenced all analyzed parameters of tested herbal infusions, leading to the development of novel products with altered and desired sensory attributes and changed chemical composition.

In the study, we demonstrated that the use of infusions obtained from mint, nettle, and blackcurrant leaves in kombucha production was a good strategy for obtaining health-beneficial polyphenol-rich beverages with increased antioxidant activity. Moreover, the results we obtained showed that bacteria and yeast, when utilizing substrates in different metabolic pathways, generated efficient metabolites, which were not only polyphenols and organic acids but also minerals. All SCOBY fermented beverages were characterized by significantly increased iron, magnesium, and calcium contents. In analyzing the relationship between the increasing content of mineral compounds and antioxidant activity due to fermentation, it should be concluded that, apart from phenolic acids, the most important element influencing the antioxidant potential was iron; in the case of fermented beverages obtained from mint and nettle, manganese; and, from blackcurrants, zinc. All of the above-mentioned microelements are components of antioxidant enzymes such as glutathione peroxidase and superoxide dismutase. However, the conducted study indicates a need for monitoring the pH and organic acid content in the new kombucha analogs to determine the maximum duration of the fermentation process and the safe amount of beverage for daily consumption.

## Figures and Tables

**Table 1:** Content of organic acids and sugar in tested beverages before and after fermentation.

Tested Beverage	Acetic Acid	Citric Acid	Succinic Acid	_D_-Glucuronic Acid	Sucrose
	[g/L]				[° Brix–g/100 mL]
Mint infusion	nd ^1^	0.23 ^a^ ± 0.041	0.04 ^b^ ± 0.011	0.01 ^b^ ± 0.001	9.8 ^a^ ± 0.00
Fermented mint infusion	5.04 ± 0.064 ^2^	0.13 ^b^ ± 0.002	0.19 ^a^ ± 0.011	0.06 ^a^ ± 0.001	8.0 ^b^ ± 0.00
Nettle infusion	nd	0.011 ^b^ ± 0.000	0.06 ^b^ ± 0.000	nd	9.6 ^a^ ± 0.00
Fermented nettle infusion	6.47 ± 0.837	0.023 ^a^ ± 0.016	0.20 ^a^ ± 0.052	0.05 ± 0.039	7.9 ^b^ ± 0.00
Blackcurrant leaves infusion	nd	0.12 ^a^ ± 0.084	0.05 ^b^ ± 0.002	nd	9.7 ^a^ ± 0.00
Fermented blackcurrant leaves infusion	3.43 ± 0.056	0.08 ^b^ ± 0.039	0.11 ^a^ ± 0.005	nd	9.0 ^b^ ± 0.00

^1^ nd = not detected; ^2^ Values are expressed as means (*n* = 3) ± standard deviations. Mean values for drinks before and after fermentation with different lowercase letters in the column are statistically different (*p* ≤ 0.05) according to Duncan’s test.

**Table 2:** Effect of fermentation on antioxidant activity of tested beverages.

Tested Beverage	PCL (µmol/mL)		Total PCL (µmol/mL)	DPPH (%)
	ACW	ACL	(ACW + ACL)	
Mint infusion	2.45 ^b^ ± 0.02 ^1^	6.70 ^b^ ± 0.11	9.14 ^b^ ± 0.13	80.2 ^a^ ± 0.05
Fermented mint infusion	5.17 ^a^ ± 0.16	8.13 ^a^ ± 0.14	13.29 ^a^ ± 0.30	74.1 ^b^ ± 0.48
Nettle infusion	0.16 ^b^ ± 0.01	0.62 ^b^ ± 0.02	0.79 ^b^ ± 0.02	15.2 ^b^ ± 0.32
Fermented nettle infusion	1.08 ^a^ ± 0.04	2.31 ^a^ ± 0.02	3.39 ^a^ ± 0.06	33.6 ^a^ ± 0.52
Blackcurrant leaves infusion	0.75 ^b^ ± 0.01	1.35 ^b^ ± 0.01	2.10 ^b^ ± 0.00	66.3 ^b^ ± 0.04
Fermented blackcurrant leaves infusion	0.93 ^a^ ± 0.00	1.64 ^a^ ± 0.01	2.57 ^a^ ± 0.01	68.05 ^a^ ± 0.77

^1^ Values are expressed as means (*n* = 3) ± standard deviations. Mean values for drinks before and after fermentation with different lowercase letters in the column are statistically different (*p* ≤ 0.05) according to Tukey’s test. PCL—photochemiluminescence method by PHOTOCHEM^®^ apparatus; ACW—antioxidant compounds soluble in water; ACL—antioxidant compounds soluble in lipids; Total PCL is the sum of results for ACW and ACL; DPPH—method based on scavenging 2,2-diphenyl-1-picrylhydrazyl radicals.

**Table 3:** Total phenolic content (TPC) and content of polyphenols (phenolic acids and flavonoids).

	Tested Beverages	Mint Infusion	Fermented Mint Infusion	Nettle Infusion	Fermented Nettle Infusion	Blackcurrant Leaves Infusion	Fermented Blackcurrant Leaves Infusion
	TPC (mg/100 mL)	64.4 ^b^ ± 1.09 ^1^	102.2 ^a^ ± 0.75	24.6 ^b^ ± 1.60	45.4 ^a^ ± 0.97	69.1 ^b^ ± 1.89	82.0 ^a^ ± 2.18
*Phenolic acids* (µg/mL)	*p*-hydroxybenzoic acid	0.01 ^b^ ± 0.00	0.02 ^a^ ± 0.00	0.01 ± 0.00	0.01 ± 0.00	n.d.	0.15 ± 0.01
	salicylic acid	<0.01 ^b^	0.07 ^a^ ± 0.01	0.01 ^b^ ± 0.00	0.13 ^a^ ± 0.02	n.d.	0.01 ± 0.00
	*m*-hydroxyphenylacetic acid	n.d.	n.d.	0.01 ± 0.00	n.d.	n.d.	n.d.
	protocatechuic acid	0.02 ^b^ ± 0.00	0.08 ^a^ ± 0.01	0.07 ^a^ ± 0.01	0.05 ^b^ ± 0.01	0.08 ± 0.00	0.08 ± 0.01
	gentisic acid	0.06 ^a^ ± 0.00	0.01 ^b^ ± 0.00	0.03 ^b^ ± 0.00	0.05 ^a^ ± 0.01	0.03 ^b^ ± 0.00	0.05 ^a^ ± 0.00
	*p*-coumaric acid	0.07 ^a^ ± 0.00	0.04 ^b^ ± 0.00	0.15 ^a^ ± 0.02	0.06 ^b^ ± 0.01	0.41 ^a^ ± 0.02	0.15 ^b^ ± 0.02
	vanilic acid	0.13 ^b^ ± 0.02	0.48 ^a^ ± 0.05	0.20 ^b^ ± 0.01	0.49 ^a^ ± 0.08	0.39 ^a^ ± 0.01	0.24 ^b^ ± 0.04
	hippuric acid	0.21 ± 0.04	0.29 ± 0.04	0.14 ^b^ ± 0.02	0.22 ^a^ ± 0.02	0.36 ^a^ ± 0.01	0.11 ^b^ ± 0.01
	caffeic acid	0.03 ^b^ ± 0.00	0.12 ^a^ ± 0.01	0.02 ^b^ ± 0.00	0.14 ^a^ ± 0.02	0.16 ^a^ ± 0.03	0.03 ^b^ ± 0.00
	ferulic acid	n.d.	1.73 ± 0.27	n.d.	0.26 ± 0.02	0.68 ± 0.02	n.d.
	syringic acid	n.d.	48.61 ± 0.59	0.58 ^b^ ± 0.06	25.19 ^a^ ± 1.15	4.06 ^b^ ± 0.10	6.40 ^a^ ± 0.23
	sinapic acid	0.15 ^b^ ± 0.01	0.43 ^a^ ± 0.07	0.13 ^b^ ± 0.01	0.25 ^a^ ± 0.02	0.25 ± 0.03	0.23 ± 0.04
	chlorogenic acid	0.01 ^b^ ± 0.00	0.02 ^a^ ± 0.00	0.16 ^a^ ± 0.00	0.01 ^b^ ± 0.00	0.30 ^a^ ± 0.03	0.09 ^b^ ± 0.00
	**sum of phenolic acids**	**0.69 ^b^ ± 0.07**	**52.09 ^a^ ± 10.08**	**1.51 ^b^ ± 0.13**	**26.86 ^a^ ± 1.36**	**6.72 ^a^ ± 0.25**	**7.54 ^a^ ± 0.36**
*Flavonoids* (µg/mL)	kaempferol	n.d.	0.01 ± 0.00	n.d.	n.d.	n.d.	n.d.
	epicatechin	0.30 ^a^ ± 0.05	0.18 ^b^ ± 0.03	0.28 ^a^ ± 0.02	0.19 ^b^ ± 0.02	0.27 ^a^ ± 0.02	0.16 ^b^ ± 0.02
	myricetin	n.d.	n.d.	n.d.	0.01 ± 0.00	0.02 ^b^ ± 0.00	0.12 ^a^ ± 0.02
	rutin	n.d.	n.d.	0.05 ± 0.00	n.d.	0.02 ± 0.00	n.d.
	**Sum of flavonoids**	**0.30 ^a^ ± 0.05**	**0.19 ^b^ ± 0.03**	**0.33 ± 0.02**	**0.20 ^b^ ± 0.02**	**0.31 ± 0.02**	**0.28 ± 0.04**
	**Sum of phenolic acids** **and flavonoids** (µg/mL)	**0.98 ^b^ ± 0.13**	**52.10 ^a^ ± 10.07**	**1.84 ^b^ ± 0.17**	**27.04 ^a^ ± 1.38**	**7.03 ± 0.29**	**7.82 ± 0.41**

^1^ Values are expressed as means (*n* = 3) ± standard deviations. Mean values for drinks before and after fermentation with different lowercase letters in the row are statistically different (*p* ≤ 0.05) according to the Duncan multiple range test; n.d.-not detected.

**Table 4:** Effect of fermentation on selected microelement contents in tested beverages (µg/100 mL).

Tested beverage	Cu	Mn	Fe	Zn
Mint infusion	2.76 ^a^ ± 0.040 ^1^	9.01 ^a^ ± 0.013	6.10 ^b^ ± 0.021	16.22 ^a^ ± 0.035
Fermented mint infusion	1.55 ^b^ ± 0.035	8.19 ^b^ ± 0.050	6.65 ^a^ ± 0.077	7.66 ^b^ ± 0.065
Nettle infusion	2.45 ^a^ ± 0.021	5.23 ^b^ ± 0.051	1.38 ^b^ ± 0.035	22.60 ^a^ ± 0.165
Fermented nettle infusion	1.20 ^b^ ± 0.020	8.25 ^a^ ± 0.040	2.92 ^a^ ± 0.035	13.62 ^b^ ± 0.015
Blackcurrant leaves infusion	2.41 ^a^ ± 0.010	12.66 ^a^ ± 0.062	1.67 ^b^ ± 0.010	12.78 ^b^ ± 0.026
Fermented blackcurrant leaves infusion	2.19 ^b^ ± 0.005	9.36 ^b^ ± 0.097	2.89 ^a^ ± 0.020	16.21 ^a^ ± 0.068

^1^ Values are expressed as means (*n* = 3) ± standard deviation. Mean values for beverages before and after fermentation with different lowercase letters in the column are statistically different (*p* ≤ 0.05) according to the Duncan multiple range test.

**Table 5:** Effect of fermentation on selected macroelement contents in tested beverages (mg/100 mL).

Tested Beverage	Mg	Ca	Na	K	P
Mint infusion	3.19 ^b^ ± 0.052 ^1^	9.01 ^b^ ± 0.016	2.19 ^b^ ± 0.012	22.16 ^a^ ± 0.050	3.85 ^a^ ± 0.031
Fermented mint infusion	4.49 ^a^ ± 0.059	9.78 ^a^ ± 0.066	2.44 ^a^ ± 0.033	18.45 ^b^ ± 0.037	3.12 ^b^ ± 0.032
Nettle infusion	3.18 ^b^ ± 0.022	13.20 ^b^ ± 0.208	2.48 ^a^ ± 0.066	13.10 ^b^ ± 0.064	5.59 ^a^ ± 0.031
Fermented nettle infusion	4.96 ^a^ ± 0.037	14.52 ^a^ ± 0.178	2.23 ^b^ ± 0.062	14.42 ^a^ ± 0.044	5.34 ^b^ ± 0.015
Blackcurrant leaves infusion	2.40 ^b^ ± 0.014	7.29 ^b^ ± 0.070	2.19 ^b^ ± 0.008	10.46 ^a^ ± 0.092	5.85 ^a^ ± 0.050
Fermented blackcurrant leaves infusion	3.79 ^a^ ± 0.043	7.81 ^a^ ± 0.042	2.44 ^a^ ± 0.043	10.20 ^b^ ± 0.065	5.11 ^b^ ± 0.042

^1^ Values are expressed as means (*n* = 3) ± standard deviation. Mean values for beverages before and after fermentation with different lowercase letters in the column are statistically different (*p* ≤ 0.05) according to the Duncan multiple range test.

## Data Availability

All of the data is contained within the article and the Supplementary Materials.

## Supplementary Materials

The following supporting information can be downloaded at https://www.mdpi.com/article/10.3390/antiox13101191/s1. Table S1: The MS data of determined phenolic acids and flavonoids; Table S2: Details on phenolic compounds quantification; Table S3. Determination range, calibration curve equation and regression coefficient of the analyzed minerals; Figure S1: LC-MS/MS chromatogram of mint infusion (a) and fermented mint infusion (b).
