# Supplementary material for: Functional Properties and Sensory Quality of Kombucha Analogs Based on Herbal Infusions
Source: Antioxidants (Basel). 2024 Sep 30;13(10):1191. doi: 10.3390/antiox13101191 (PMC11505365; doi:10.3390/antiox13101191)
Supplement: Supplementary file 1 [file antioxidants-13-01191-s001.zip › antioxidants-3205454-supplementary.pdf]

**Table S1: The MS data of determined phenolic acids and flavonoids.**

| Compounds | $R_t$ (min) | $[M]^-$ (*m/z*) | MS/MS (*m/z*) | MS/MS product ion spectrum |
|---|---|---|---|---|
| Caffeic acid | 2.41 | 179 | 135/107 | 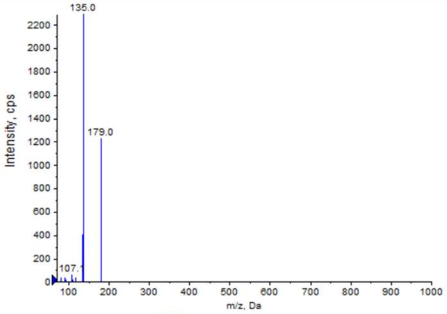 |
| Chlorogenic acid | 2.21 | 353 | 191/179/147 | 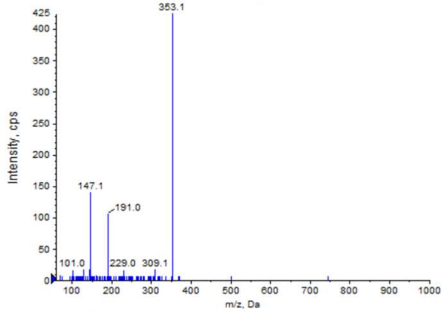 |
| *p*-Coumaric acid | 2.51 | 163 | 119/93 | 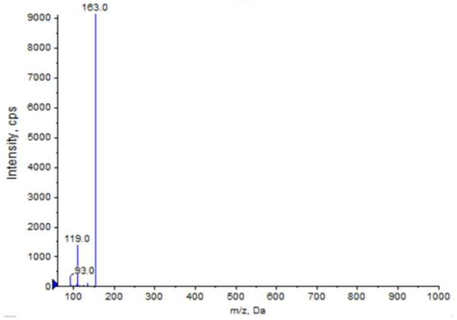 |

| | | | | |
|---|---|---|---|---|
| Ferulic acid | 2.52 | 193 | 178/134 | 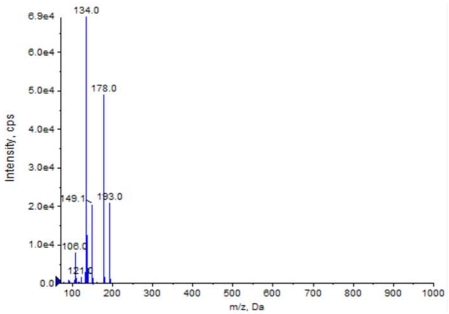 |
| *p*-Hydroxybenzoic acid | 2.30 | 137 | 93/65 | 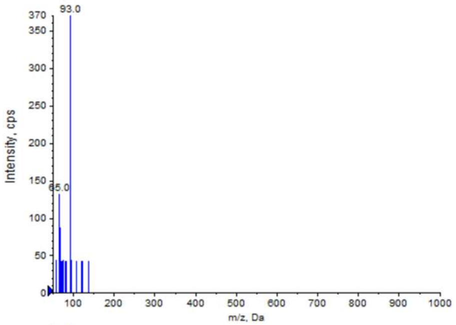 |
| *m*-Hydroxyphenylacetic acid | 2.41 | 151 | 135/107/93 | 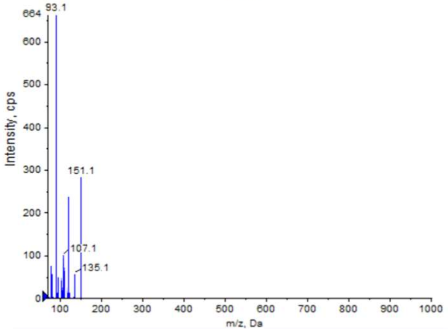 |

| | | | | |
|---|---|---|---|---|
| Protocatechuic acid | 1.91 | 153 | 91/81 | 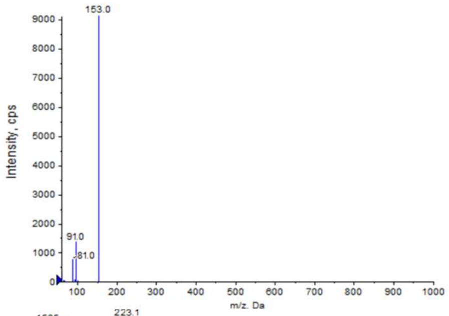 |
| Sinapic acid | 2.55 | 223 | 208/179/164 | 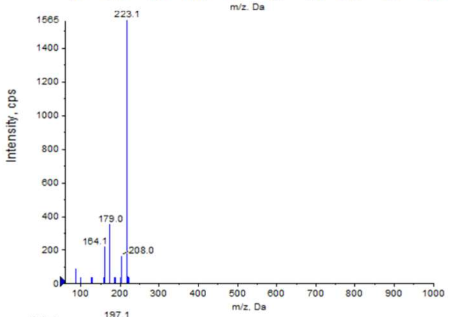 |
| Syringic acid | 2.45 | 197 | 182/153 | 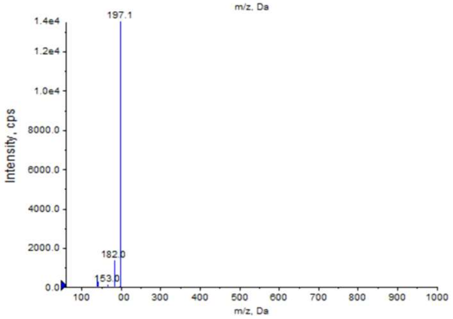 |

| | | | | |
|---|---|---|---|---|
| Vanillic acid | 2.27 | 167 | 123/108/91 | 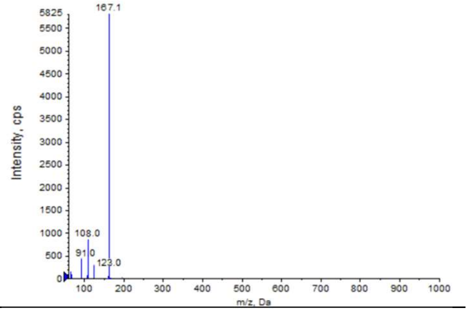 |
| Epicatechin | 2.33 | 289 | 245/203/109 | 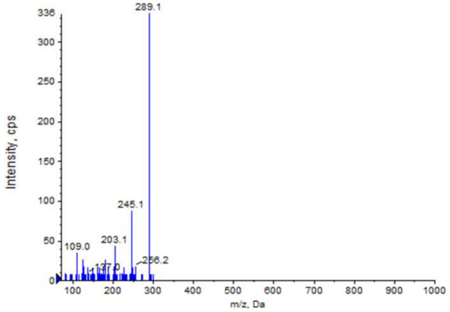 |
| Kaempferol | 2.92 | 285 | 185/93 | 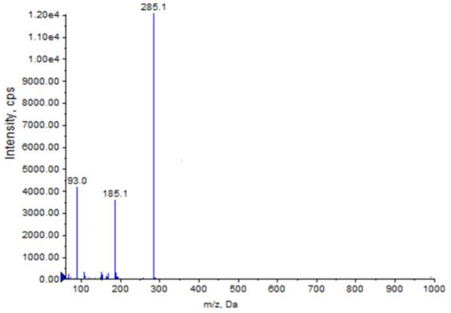 |

| | | | | |
|---|---|---|---|---|
| Rutin | 2.34 | 609 | 463/301 | 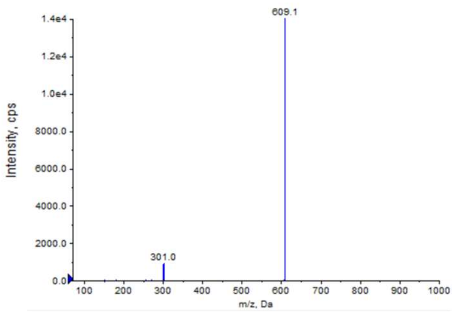 |



**Table S2: Details on phenolic compounds quantification.**

| | **List of compounds** | Curve equation | Conc. Range [mg/mL] | $R^2$ |
|---|---|---|---|---|
| *Phenolic acids* | *p*-hydroxybenzoic acid | *y=3.07e+10x* | 0.0001-0.002 | 0.979 |
| | salicylic acid | *y=4.03 e +10x* | 0.000003-0.0006 | 0.998 |
| | *m*-hydroxyphenylacetic acid | *y=3.13 e +9x* | 0.0001-0.001 | 0.997 |
| | protocatechuic acid | *y=2.57 e +10x* | 0.000003-0.0007 | 1.000 |
| | gentisic acid | *y=8.87 e +9x* | 0.00001-0.002 | 0.998 |
| | *p*-coumaric acid | *y=7.72 e +9x* | 0.00003-0.0003 | 0.997 |
| | vanilic acid | *y=4.85 e +8x* | 0.00005-0.05 | 0.999 |
| | hippuric acid | *y=8.18 e +8x* | 0.00001-0.003 | 1.000 |
| | caffeic acid | *y=8.57 e +9x* | 0.00006-0.001 | 0.993 |
| | ferulic acid | *y=1.46 e +9x* | 0.000006-0.001 | 0.985 |
| | syringic acid | *y=8.10 e +7x* | 0.00003-0.003 | 1.000 |
| | sinapic acid | *y=5.04 e +8x* | 0.000002-0.0004 | 0.989 |
| | chlorogenic acid | *y=6.02 e +10x* | 0.000001-0.0002 | 0.998 |
| *Flavonoids* | kaempferol | *y=4.37 e +9x* | 0.00001-0.0001 | 0.997 |
| | epicatechin | *y=8.93 e +8x* | 0.0001-0.005 | 0.999 |
| | myricetin | *y=1.18 e +10x* | 0.0000009-0.009 | 0.994 |
| | rutin | *y=2.51 e +9x* | 0.0000005-0.0005 | 0.990 |

**Table S3. Determination range, calibration curve equation and regression coefficient of the analyzed minerals.**

| Mineral | Determination range of the calibration curve (µg/ml) | Calibration curve equation (y=ax+b) | Regression coefficient ($R^2$) |
|---|---|---|---|
| Cu | 0,05-0,8 | y=0,15098x+0,00007 | 0,99999 |
| Mn | 0,05-1,6 | y=0,0708x+0,0006 | 0,9998 |
| Fe | 0,2-1,6 | y=0,2381x+0,0062 | 0,9992 |
| Zn | 0,05-0,8 | y=0,366x+0,0078 | 0,9984 |
| Mg | 0,05-0,8 | y=1,1205x+0,058 | 0,9953 |
| Ca | 0,5-4,0 | y= 0,0455x+0,0055 | 0,9995 |
| Na | 0,5-4,0 | y=22,284+11,985 | 0,9982 |
| K | 2,0-20,0 | y=4,6449x+9,2497 | 0,9891 |
| P | 0,4-2,0 | y=0,0037x+0,0005 | 0,9995 |

a.

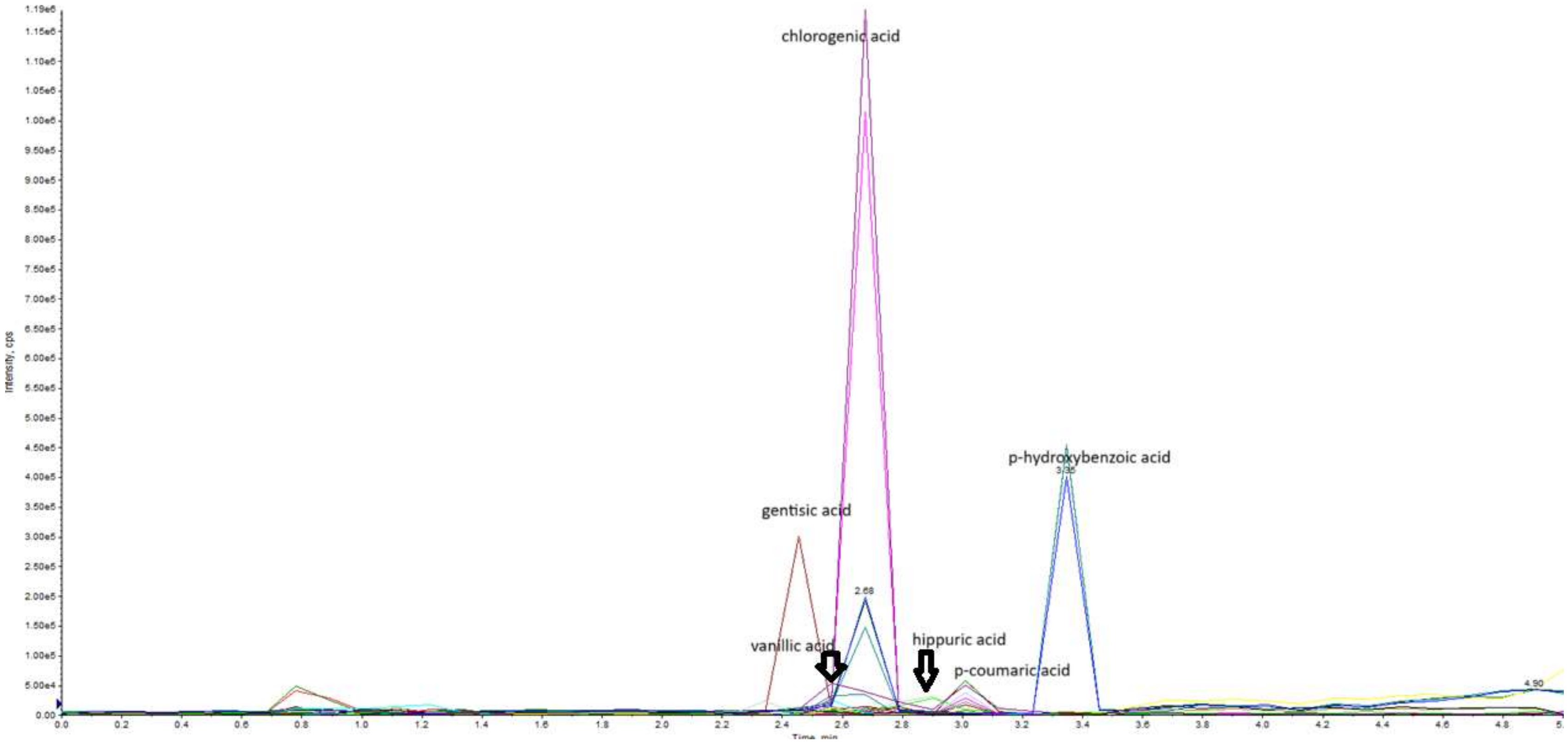
chlorogenic acid
p-hydroxybenzoic acid
gentisic acid
vanillic acid
hippuric acid
p-coumaric acid
2.68
3.35
4.90
Intensity, cps
Time, min

b.

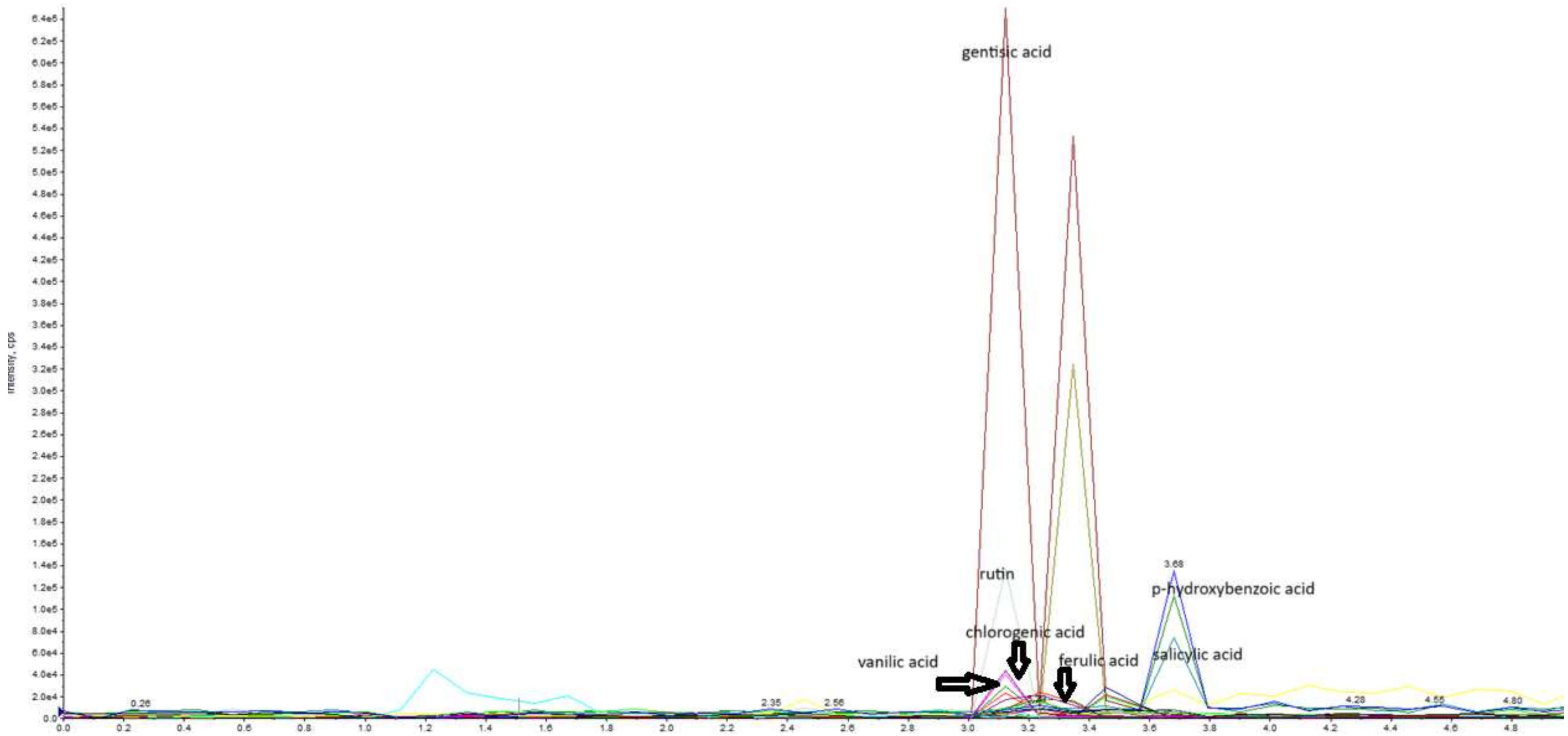


Figure S1. LC-MS/MS chromatogram of mint infusion (a) and fermented mint infusion (b).
